# Effect of Dietary Coenzyme Q10 Plus NADH Supplementation on Fatigue Perception and Health-Related Quality of Life in Individuals with Myalgic Encephalomyelitis/Chronic Fatigue Syndrome: A Prospective, Randomized, Double-Blind, Placebo-Controlled Trial

**DOI:** 10.3390/nu13082658

**Published:** 2021-07-30

**Authors:** Jesús Castro-Marrero, Maria Jose Segundo, Marcos Lacasa, Alba Martinez-Martinez, Ramon Sanmartin Sentañes, Jose Alegre-Martin

**Affiliations:** 1 ME/CFS Research Unit, Division of Rheumatology, Vall d’Hebron Hospital Research Institute, Universitat Autònoma de Barcelona, 08035 Barcelona, Spain; rsanmartin@vhebron.net (R.S.S.); jalegre@vhebron.net (J.A.-M.); 2 Clinical Research Department, Vitae Health Innovation, Montmeló, 08160 Barcelona, Spain; msegundo@vitae.es; 3 Department of Education, Generalitat de Catalunya, Sabadell, 08202 Barcelona, Spain; marcos.lacasa@gmail.com; 4 Medical Oncology Clinical Trials Office, Vall d’Hebron Institute of Oncology, Vall d´Hebron University Hospital, Universitat Autònoma de Barcelona, 08035 Barcelona, Spain; albamartinez@vhio.net

**Keywords:** chronic fatigue syndrome, coenzyme Q10, myalgic encephalomyelitis, mitochondria, nonrestorative sleep, NADH, quality of life

## Abstract

Myalgic encephalomyelitis/chronic fatigue syndrome (ME/CFS) is a complex, multisystem, and profoundly debilitating neuroimmune disease, probably of post-viral multifactorial etiology. Unfortunately, no accurate diagnostic or laboratory tests have been established, nor are any universally effective approved drugs currently available for its treatment. This study aimed to examine whether oral coenzyme Q10 and NADH (reduced form of nicotinamide adenine dinucleotide) co-supplementation could improve perceived fatigue, unrefreshing sleep, and health-related quality of life in ME/CFS patients. A 12-week prospective, randomized, double-blind, placebo-controlled trial was conducted in 207 patients with ME/CFS, who were randomly allocated to one of two groups to receive either 200 mg of CoQ10 and 20 mg of NADH (*n* = 104) or matching placebo (*n* = 103) once daily. Endpoints were simultaneously evaluated at baseline, and then reassessed at 4- and 8-week treatment visits and four weeks after treatment cessation, using validated patient-reported outcome measures. A significant reduction in cognitive fatigue perception and overall FIS-40 score (*p* < 0.001 and *p* = 0.022, respectively) and an improvement in HRQoL (health-related quality of life (SF-36)) (*p* < 0.05) from baseline were observed within the experimental group over time. Statistically significant differences were also shown for sleep duration at 4 weeks and habitual sleep efficiency at 8 weeks in follow-up visits from baseline within the experimental group (*p* = 0.018 and *p* = 0.038, respectively). Overall, these findings support the use of CoQ10 plus NADH supplementation as a potentially safe therapeutic option for reducing perceived cognitive fatigue and improving the health-related quality of life in ME/CFS patients. Future interventions are needed to corroborate these clinical benefits and also explore the underlying pathomechanisms of CoQ10 and NADH administration in ME/CFS.

## 1. Introduction

Myalgic encephalomyelitis, commonly referred to as chronic fatigue syndrome (ME/CFS), is a serious, complex, and chronic multisystem illness of unknown etiology, often triggered by a persistent viral infection (for this reason, it is also known as post-viral fatigue syndrome). It is characterized by unexplained and persistent post-exertional fatigue, which is unrelieved by rest and made worse by physical and mental effort, along with other core symptoms such as the disruption of cognitive, immunometabolic, autonomic, and neuroendocrine pathways [1]. It affects as many as 17 to 24 million people worldwide, and its prevalence is expected to more than double by 2030 [2]. The clinical presentation and symptom frequency and severity may vary between patients, but in general, the illness reduces overall quality of life and social, occupational, and personal activity; indeed, some patients are even bedridden or housebound [3]. Diagnosis of ME/CFS is based on patients’ core symptoms according to a consensus of four case criteria developed over the past 25 years, which have been applied both in research and in clinical practice: the 1994 CDC/Fukuda definition [4], the 2003 Canadian Consensus Criteria (also known as CCC) [5], the 2011 International Consensus Criteria (ICC) [6], and, most recently, the 2015 IOM Expert Criteria for Systemic Exertion Intolerance Disease (SEID) [7]. In the clinical diagnosis of ME/CFS, it is important to evaluate the disabling fatigue perception, sleep problems, and health-related quality of life using validated questionnaires such as the fatigue impact scale (FIS-40) [8], Pittsburg sleep quality index (PSQI) [9], and Short Form Health Survey (SF-36) [10]. Unfortunately, at present, there are no commercially available diagnostic tests, no specific lab biomarkers, and no targeted FDA-approved drugs for ME/CFS [11].

Coenzyme Q10 (CoQ10) and the reduced form of nicotinamide adenine dinucleotide (NADH) are key components of the electron transport chain responsible for mitochondrial ATP production, which decreases free radical generation, and, in their reduced forms, they act as powerful antioxidants. CoQ10 and NADH levels and redox status have been shown to be disturbed in ME/CFS. Strong evidence has emerged that mitochondrial dysfunction, disturbed immunometabolism, and increased oxidative stress play a pivotal role in the pathogenesis of numerous illnesses, providing a robust scientific rationale for testing potential “nutraceuticals” that target these processes [12].

As chronic fatigue is a key diagnostic symptom for ME/CFS, it has been suggested that energy metabolism may be disrupted in a subset of patients with ME/CFS. Mitochondrial dysfunction has been considered as a possible underlying pathomechanism of the illness, based on the structural and functional changes reported vis-a-vis healthy controls [12]. Long-chain polyunsaturated fatty acid, prostaglandin and amino acid metabolism, and inefficient ATP biosynthesis have been suggested as consequences of ME/CFS due to perturbed mitochondrial bioenergetic metabolism [13]. Due to the potential role of the mitochondria in ME/CFS, mitochondrial-targeting nutraceutical interventions have been used to assist in improving patient outcomes such as fatigue and their health-related quality of life [14], including CoQ10, NADH, and n-Acetyl-L-carnitine as part of their treatment regime [15,16,17,18]. These treatments are administered either alone or in combination with a cocktail of other nutraceutical and/or pharmaceutical-based products.

Over the last decade, a growing body of data has supported the potential clinical use of dietary CoQ10 supplementation in health and chronic illnesses [13,19,20,21,22,23,24,25].

Previously, in a proof-of-concept study, our group found a trend towards improvement over time in fatigue perception, response to physical exercise, and biochemical parameters of oxidative stress and mitochondrial metabolism (ATP content, lipid peroxidation, citrate synthase activity, levels of CoQ10 and NADH) in peripheral blood mononuclear cells after CoQ10 plus NADH administration [26,27,28]. There is evidence of improved mitochondrial respiration following prolonged CoQ10 supplementation [29,30], via mechanisms involving the upregulation of enzymes that regulate fatty acid transport into organelles and also the stimulation of mitochondrial biogenesis [31,32]. Among individuals suffering from a wide range of illnesses, researchers have also reported reductions in reactive oxygen and nitrogen species levels (ROS/RNS), protein carboxylation, DNA damage, protein nitration, and lipid peroxidation following CoQ10 supplementation [33,34]. Other in vivo evidence of reduced levels of oxidative and nitrosative stress following CoQ10 supplementation includes downregulation of iNOS enzyme and NF-kB [35,36]. Coenzyme Q10 has been shown to reduce markers of systemic inflammation in several metabolic, autoimmune, and neurological disorders [37,38]. As for NADH, it is known to facilitate the generation of intracellular ATP [39], and in several studies, it has shown a higher response rate during the first trimester of treatment compared to control subjects [40]. In another proof-of-concept cohort study, our group showed that NADH managed to improve anxiety/depression symptoms and maximum heart rate in ME/CFS following a two-day consecutive challenge test (2-day CPET), findings that may imply an improvement in the oxygen supply to the skeletal muscle and to the brain in individuals with ME/CFS [41].

Data on the potential therapeutic effects of CoQ10 and NADH co-supplementation on fatigue, pain, and sleep impairments in individuals with ME/CFS are scarce. The current study was conducted to evaluate the clinical effects of CoQ10 plus NADH supplementation on fatigue perception, nonrestorative sleep problems, and HRQoL in people with ME/CFS, due to a previous pilot study in which no significant differences were found for chronic pain and sleep quality in ME/CFS.

## 2. Materials and Methods

### 2.1. Participants

**Figure 1:**
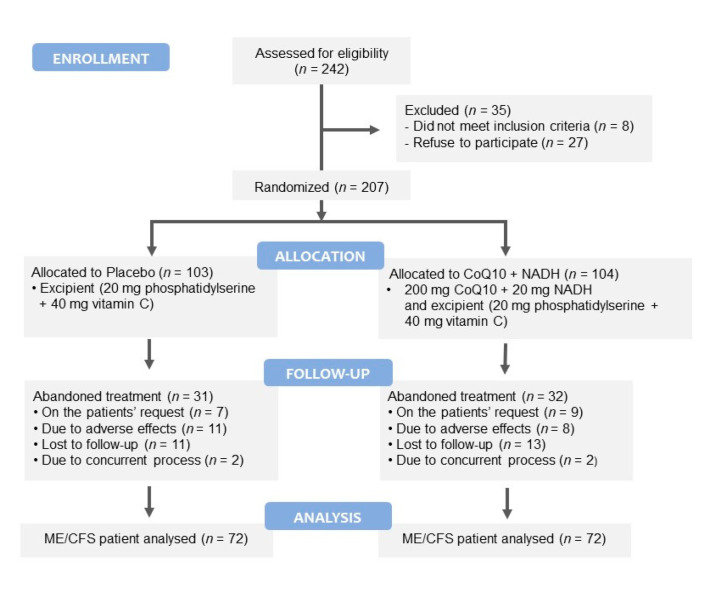
Consolidated Standards of Reporting Trials (CONSORT) flow diagram illustrating the steps of screening, enrollment, assignment, and follow-up of the study participants.

A 12-week, single-center, randomized, double-blind, placebo-controlled trial was conducted in 242 Caucasian ME/CFS patients consecutively recruited from a single outpatient tertiary referral center (ME/CFS Clinical Unit, Vall d’Hebron University Hospital, Barcelona, Spain) from January 2018 to December 2019. Figure 1 shows a flowchart of the participants prior to analysis. Patients were eligible for the study if they were female, aged 18 years or older, had a confirmed diagnosis of ME/CFS according to the 1994 CDC/Fukuda case definition [4], and provided signed written informed consent. Exclusion criteria included any active medical condition that explained the chronic fatigue (untreated hypothyroidism, sleep apnea, narcolepsy, medication side effects, and iron deficiency anemia), previous diagnosis not unequivocally resolved (chronic hepatitis, malignancy), past or current neuropsychiatric disorders (major depressive disorder with psychotic or melancholic features, bipolar disorder, schizophrenia, delusional disorder, dementias, anorexia nervosa, bulimia nervosa), and participation in another clinical trial of the same or different nature within 30 days prior to study inclusion; inability (in the opinion of the investigator) to follow the instructions or to complete the treatment satisfactorily; failure to provide signed informed consent; consumption of certain drugs/supplements that might influence outcome measures in the last 90 days or whose withdrawal might be a relevant problem, anticoagulant treatment, pregnancy or breast-feeding, smoking, alcohol intake or substance abuse, obesity (BMI > 30 kg/m^2^), and hypersensitivity to CoQ10 or NADH. Patients with missing data from the follow-up visits to baseline were considered to have dropped out.

### 2.2. Intervention

Of the 242 eligible ME/CFS participants screened, 35 were excluded (eight who did not meet the inclusion criteria, and 27 who refused to participate). The remaining 207 participants were allocated to treatment by an independent investigator not otherwise involved in the intervention, using the result of a list of random numbers generated by a computer program. The participants were randomly assigned in a double-blind fashion in a 1:1 ratio to receive either 200 mg of CoQ10 plus 20 mg of NADH (*n* = 72) or a matching placebo (*n* = 72), in four capsules daily for eight weeks; all participants also received their standard therapy for the disease. The CoQ10 was used in combination with NADH, in light of reports that they may have synergistic antioxidant effects and that NADH may enhance CoQ10 absorption [26,27].

Safety information obtained at all study visits included data on adverse events, data from clinical laboratory tests, vital signs, along with the results of the general physical examination. Adverse events, including serious ones, were reviewed throughout the trial by the independent medical monitor, the steering committee, and the independent data and safety monitoring board. Provision was made for investigator-initiated temporary or permanent dose reductions or suspensions due to adverse effects.

During the study, 19 subjects dropped out due to adverse effects: eight in the active group (five epigastralgias, and three dizziness), and 11 in the placebo group (four epigastralgias, five dizziness, one diarrhea, and one muscle spasm). Twenty-four patients were lost to follow-up (13 in the experimental group and 11 in the placebo group). Four discontinued the study due to the presence of a concurrent process (one case of hypotensive treatment in each group, one case of anemia in the experimental group, and one case of foot trauma in the placebo group). Finally, 16 patients withdrew at their own request (nine cases in the experimental group and seven within in the placebo group). The remaining 144 cases of ME/CFS (70%, 72 patients in each treatment group) completed all the study protocol procedures and were included in the overall analysis of outcome measures (see Figure 1).

### 2.3. Product Tested

Patients randomized to the CoQ10 plus NADH experimental group received four enteric-coated tablets daily consisting of active ingredients (50 mg of CoQ10 and 5 mg of NADH) and excipients (20 mg of phosphatidylserine and 40 mg of vitamin C). Patients randomized to placebo received supplementation comprising four enteric-coated tablets daily containing only excipients. Both experimental and placebo tablets were identical in size, color, opacity, shape, presentation, and packaging. All tablets were manufactured and donated by Vitae Health Innovation, S.L. (Montmeló, Barcelona, Spain). The study pharmacist recorded all treatments supplied on the medication-dispensing forms along with the original script.

### 2.4. Study Design and Procedures

**Figure 2:**
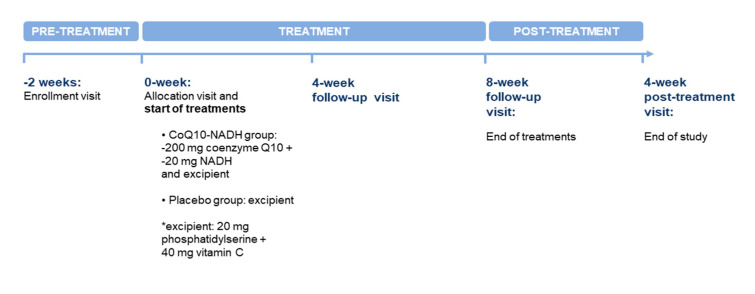
Summary of the study schedule at each visit during the clinical trial.

This unicenter, randomized, double-blind, placebo-controlled cohort study design was conducted to evaluate the clinical benefits of oral CoQ10 plus NADH administration on fatigue perception, unrefreshing sleep problems, and HRQoL in ME/CFS. Clinical visits throughout the study are detailed in Figure 2, which also describes the trial design in both groups. After a verbal description of the study, all participants gave written consent prior to commencement and received no compensation for their participation. Patients were evaluated at baseline, and then at four and eight weeks of treatment, and finally four weeks after the end of treatment by the site investigator. Changes in symptoms were assessed through validated self-reported questionnaires completed by participants under the supervision of two trained investigators (J.C.-M. and J.A.-M.). Compliance was checked through medication logs. Use of concomitant medications was tracked at all follow-up visits. The study protocol was reviewed and approved by the local institutional review board at the participating site (Clinical Research Ethics Committee, Vall d’Hebron University Hospital, Barcelona, Spain; protocol code: VITAE-2015, approved on 24 April 2015). The study protocol was conducted in accordance with the guidelines of the Declaration of Helsinki, and with the current Spanish regulations on clinical research and the standards of good clinical practice of the European Union. It also followed the Consolidated Standards of Reporting Trials (CONSORT) guidelines. The current clinical trial was registered on https://clinicaltrials.gov as NCT03186027 (accessed on 29 June 2017).

### 2.5. Primary Endpoint

The prospectively defined main outcome measure was the change in self-reported fatigue scores assessed using the validated Fatigue Impact Scale (FIS-40) from baseline to the final study visit held three months later. The study was powered to detect a 3-point difference between active treatment group and placebo. The FIS-40 comprises 40 items divided into three domains that describe how perceived fatigue impacts upon cognitive (10 items), physical (10 items), and psychosocial functioning (20 items) over the previous four weeks. Each item is scored from 0 (no fatigue) to 4 (severe fatigue). The total FIS-40 score is calculated by adding together responses from the 40 questions (score range 0–160). Higher scores indicate more functional limitations due to severe fatigue [8].

### 2.6. Secondary Endpoints

The secondary outcome measures included changes in sleep disturbances and health-related quality of life through validated self-reported PSQI and HRQoL questionnaires, respectively.

#### 2.6.1. Pittsburgh Sleep Quality Index

Sleep disturbances were assessed through the self-administered 19-item Pittsburgh Sleep Quality Index (PSQI) questionnaire. Scores are obtained on each of seven domains of sleep quality: subjective sleep quality, sleep latency, sleep duration, habitual sleep efficiency, sleep perturbations, use of sleeping medication, and daytime dysfunction. Each component is scored from 0 to 3 (0 = no sleep problems and 3 = severe sleep problems). The global PSQI score ranges from 0 to 21 points, with scores of ≥5 indicating poorer sleep quality [9].

#### 2.6.2. The 36-Item Short Form Health Survey

The 36-item Short Form Health Survey (SF-36) was used to assess health-related quality of life. The SF-36 is a broadly-based self-reported survey of health-related physical and mental functioning status in ME/CFS and other chronic conditions. It assesses functioning on eight subscales including domains of physical functioning, physical role, bodily pain, general health, social functioning, vitality, emotional role, and mental health, and two additional general subscales covering the physical and mental health domains, rated on a scale from 0 to 100. Lower scores indicate a more negative impact of an individual’s health on functioning [10].

### 2.7. Power and Sample Size Estimation

The sample size was estimated based on data from a previous clinical trial of CoQ10 and NADH supplementation in people with ME/CFS [28]. Allowing for an estimated early drop-out rate of 20%, and accepting an alpha risk of 5% and a beta risk of 20% (two-sided test), a total of 121 subjects in each group was considered necessary to find statistically significant differences between groups, expected to be 10% in the placebo group and 25% in the experimental group (https://www.imim.es/ofertadeserveis/software-public/granmo/, accessed on 12 June 2018).

### 2.8. Monitoring of Compliance and Adverse Events

All participants were asked to return any remaining study product after the intervention. Adherence was measured by calculating all remaining tablets. Participants who did not take the supplement on more than two non-consecutive days or consecutive days were considered non-compliant (*n* = 0). All adverse events following administration of study product intake were monitored until the end of the study. No serious adverse events or suspected adverse reactions (e.g., events resulting in death, immediately life-threatening events, medically significant events for any reason, events requiring or prolonging hospitalization or resulting in persistent or significant disability/incapacity) were reported during or after the end of the study.

### 2.9. Statistical Analysis

A descriptive statistical analysis was carried out for all study variables. Results are shown as means ± standard deviation (SD). A Kolmogorov–Smirnov test was used to determine the distribution and normality of data for both groups. Continuous variables were defined by the number of valid cases and expressed as mean ± SD. Categorical variables were described using absolute and relative frequencies of each category over the total of valid values. Differences in comparison analysis between the two groups were assessed using Student’s *t*-test (paired data *t*-test using the value of the patient as a control) for continuous variables and the Chi-square test for categorical variables. For all comparisons, a two-sided level of statistical significance of 0.05 was considered. All analyses were performed on the data set using all available information with intention-to-treat (ITT) criteria. An analysis of covariance (one-way ANCOVA) was performed using the changes in overall FIS-40 score from baseline to the last follow-up visit of the study as the primary outcome measure, and entering the overall FIS-40, PSQI, and HRQoL scores at baseline as continuous predictors and the enrolling investigator and treatment assigned as categorical predictors. The statistical analysis observed the ICH-E9 guidelines as well as all the rules of good clinical practice. All data were analyzed using the SAS program, version 9.4 (Statistical Analysis System Institute Inc., Cary, NC, USA).

## 3. Results

### 3.1. Participants’ Characteristics at Baseline

Table 1 shows the demographic and clinical characteristics of the study participants. No statistically significant differences were found in terms of demographic and clinical parameters between the study groups at baseline. No statistically significant differences were observed in the standard drug therapy used within the participants’ treatment groups. The following are the most frequent concomitant drugs (more than 90%) that subjects were taking during the study: anticonvulsants (gabapentin), tricyclic antidepressants (duloxetine), anxiolytics/hypnotics (benzodiazepines on demand), NSAIDs (ibuprofen on demand), and opioids (tramadol).

### 3.2. Changes in Fatigue Perception among Participants

Table 2 shows participants’ scores for fatigue perception over the course of the clinical study. Perception of cognitive fatigue (as indicated by the change in the cognitive domain from FIS-40) improved significantly at the 4- and 8-week visits from baseline within the active group (*p* = 0.005 and *p* = 0.010, respectively). The psychosocial domain showed a nominal improvement in the active group at the 4-week visit, though without reaching statistical significance (*p* = 0.053).

In the CoQ10 + NADH group, the total FIS-40 score significantly decreased at the 4-week visit from baseline (*p* = 0.022). However, this improvement was not significant at the follow-up visits from baseline (8 weeks and 4 weeks post-treatment; *p* = 0.089 and *p* = 0.071, respectively). FIS-40 domain scores evolved in parallel between groups over the course of the study.

### 3.3. Changes in Sleep Quality and Health-Related Quality of Life after CoQ10 Plus NADH Supplementation in the Study Population

#### 3.3.1. Pittsburgh Sleep Quality Index

Table 3 displays participants’ sleep quality scores assessed using the PSQI questionnaire. In the between-group comparison analysis, statistically significant differences were displayed for sleep duration at 4-week follow-up visit from baseline in the experimental group (*p* = 0.018). Moreover, in the within-experimental group comparison analysis, we found statistically significant differences for the PSQI domains from baseline over time (all *p*-values < 0.05).

#### 3.3.2. The Short Form 36-Item Health Survey

Table 4 shows participants’ scores on the SF-36 questionnaire. Physical role functioning, general health perception, vitality, social role functioning, emotional role functioning, and mental health status domains did not show any differences in the between-group comparison analysis.

The physical functioning showed better scores in the CoQ10 plus NADH group; statistically significant improvements were observed at both visits from baseline during treatment (4-week and 8-week visits; *p* = 0.036 and *p* = 0.001, respectively). The bodily pain domain improved at the 4-week visit from baseline (*p* = 0.043). In the placebo group, a reduction in vitality was observed 4 weeks after treatment discontinuation (*p* = 0.042).

### 3.4. Clinical Safety and Tolerability Evaluation

With regard to safety data, few adverse effects have been associated with CoQ10 and NADH supplementation [18,39] in other populations. In our study of ME/CFS patients, no relevant treatment-related adverse events were recorded. Our findings demonstrate that oral CoQ10 plus NADH supplementation for eight weeks was safe and potentially well-tolerated by the participants.

## 4. Discussion

The use of nutritional interventions as antioxidant supplements in order to reduce increased oxidative stress in ME/CFS patients remains a controversial issue [14]. CoQ10 and NADH are two possible candidates for use in the treatment of ME/CFS, and perhaps in other illnesses involving chronic fatigue in which oxidative stress plays a significant role, for at least three main reasons. First, CoQ10 and NADH are bioenergetic cofactors with the potential to boost mitochondrial function. Second, CoQ10 and NADH are powerful free radical scavengers that can mitigate the lipid peroxidation and DNA damage caused by oxidative stress; third, they have powerful antioxidant properties [17].

To the best of our knowledge, this is the first RCT to investigate the beneficial effects of oral CoQ10 plus NADH supplementation on perceived fatigue, nonrestorative problems, and HRQoL in a substantial number of people with ME/CFS. Our results showed that the combination of CoQ10 plus NADH had a positive effect on the perception of fatigue, sleep quality, and HRQoL in ME/CFS. This was highlighted in the improvement in scores observed in the intragroup analysis, although no differences were found in the intergroup analysis.

Coenzyme Q10 and NADH deficiencies have also been described in individuals with ME/CFS and fibromyalgia [26,42], and it has suggested that serum NADH levels are directly correlated with serum CoQ10 concentrations in these patients. CoQ10, also known as ubiquinone, is a fat-soluble, vitamin-like benzoquinone compound that is endogenously synthesized from tyrosine in the human body. It is an essential component in the metabolism of all cells, and a CoQ10 deficiency is linked to the pathogenesis of a range of chronic disorders. It is a strong antioxidant agent that confers resistance to the mitochondrial damage induced by oxidative and nitrosative stress, and it also serves as an anti-inflammatory agent, affecting ATP synthesis and nitric oxide preservation [14]. Chronically activated immune responses and nitrosative and oxidative stress in ME/CFS patients induce disorders such as brain hypoperfusion/hypometabolism, neuroinflammation, DNA damage, mitochondrial dysfunction, secondary autoimmune responses directed against disrupted proteins and lipid membrane components, and dysfunctional intracellular signaling pathways [15].

The administration of NADH alone [43] and in combination with CoQ10 [27,28] has been shown to reduce fatigue in people with ME/CFS. However, although those studies reported beneficial effects, none of them reported the baseline dietary intake characteristics, and this may have influenced the results.

Alongside chronic fatigue, the most hallmark symptoms of ME/CFS are intolerance to physical/mental exercise (post-exertional malaise), alterations in concentration/memory, nonrestorative sleep, orthostatic intolerance, and generalized chronic pain [1]. Given the natural course towards chronicity in ME/CFS, we consider the results obtained in our study to be important. We confirm that CoQ10 plus NADH supplementation has a positive effect on the perception of cognitive fatigue, with significant improvements at 4- and 8-week visits (*p* < 0.001) compared to baseline. Likewise, our dietary nutraceutical combination may have influenced the overall FIS-40 domain score—reducing it at the 4-week visit from baseline (*p =* 0.022), although the difference was not significant at the end of treatment visit (8-weeks vs. baseline, *p* = 0.08)—and also the psychological domain, which showed a marked improvement at the 4-week visit (though also without reaching significance vs. baseline, *p* = 0.05) within the experimental group. In previous research carried out by our group and others, with nutraceutical interventions to assess mitochondrial dysfunction in ME/CFS, fatigue was the primary endpoint in eight out of nine studies [28,43,44,45,46,47,48,49]. Of these studies, five found significant differences in fatigue levels following an intervention [28,46,47,48,49]; however, other authors reported no significant changes in fatigue levels in ME/CFS [44,45]. The discrepancies between these studies may be due to the heterogeneity of the ME/CFS population (i.e., sociodemographic and clinical characteristics of the patients, diagnostic criteria used, presence of different comorbid health conditions, and pharmacological and non-pharmacological treatments administered), the small sample size, and the short study duration, among other factors.

In experiments on rodents, CoQ10 treatment improved cognitive deficits by modulating mitochondrial functions and was effective in protecting against cognitive impairments and neurodegeneration in experimental animal models [50,51]. Fukuda et al. [44] suggested that ubiquinol-10 supplementation may improve cognitive impairments by modulating energy reserves in ME/CFS, and Dumont et al. [52] reported that CoQ10 administration improved cognitive performance in a transgenic mouse model of Alzheimer’s disease.

Clinical trials have shown that oral replacement supplements of CoQ10 plus/or NADH significantly reduce fatigue perception and other symptoms associated with chronic diseases. Several studies in people with fibromyalgia [42] have reported improvements in chronic pain, fatigue, and HRQoL, and studies in other neuroimmune conditions such as remitting–relapsing multiple sclerosis have demonstrated the effectiveness of CoQ10 administration in reducing in vivo inflammation and oxidative stress, and in improving fatigue and anxiety/depression symptoms [52,53]. Based on these findings, new potential therapeutic strategies are now urgently needed [54].

Our intragroup comparison analysis found that ME/CFS patients supplemented with CoQ10 plus NADH showed significant increases in the physical function domain of the SF-36 at both follow-up visits. This result is of interest, because our review of the scientific literature revealed improvements in health-related quality of life after CoQ10 administration in other related chronic conditions such as fibromyalgia and Gulf War Syndrome [55,56], but not in ME/CFS.

In the broad group of neurological manifestations of ME/CFS, the component of generalized pain stands out, both in the context of ME/CFS and in the case of comorbid fibromyalgia [57]. In our study, the CoQ10 plus NADH supplementation induced an improvement on the bodily pain component of the SF-36 questionnaire at the 4-week treatment visit from baseline in the study participants. A previous study by our group to evaluate chronic pain using the McGill Pain questionnaire found no changes in pain from ME/CFS participants [28].

In patients with ME/CFS, nonrestorative sleep leads to a significant alteration in sleep quality [58]. Our results show that the CoQ10 plus NADH administration improved the perception of sleep quality, demonstrating new findings that differ from the aforementioned earlier study by our group [28], which also used the same validated PSQI questionnaire. On the contrary, a study in veterans with Gulf War Syndrome, which bears similarities to ME/CFS, found that only CoQ10 supplementation improved symptoms of physical function but had no beneficial effect on sleep quality [55].

Previous studies investigating the beneficial clinical effects of CoQ10 supplementation in heart failure (HF) have been conducted over the last few years. The findings have reported an improvement in bioenergetic and functional levels and endothelial function in these HF patients [59,60].

Previous reports have suggested that dietary supplements such as CoQ10 or NADH are safe and well-tolerated [18]. Our results confirm these observations and suggest that moderate doses of these molecules can be safely added to conventional therapy in ME/CFS.

### Strengths and Limitations

This study has some limitations that must be considered. Firstly, the primary endpoint, the perception of fatigue recorded through the FIS-40 questionnaire, is a subjective parameter. In future work, objective physiological and biological parameters might be used, such as the maximum oxygen consumption and lactate/glucose levels during and after performing a cardiopulmonary challenge test (2-day consecutive CPET). Secondly, there were limitations associated with the study design, research setting, and participants’ selection. The fact that all patients were Caucasian ME/CFS women and were recruited from a single tertiary referral center may have increased the proportion of seriously ill patients, and we must be cautious when generalizing these results to other populations in other healthcare settings such as primary care or the general population. Thirdly, the lack of statistical differences found between groups may have been due to the placebo effect (phosphatidylserine and vitamin C as excipients). Finally, the doses and follow-up timing of the intervention were pre-established, and so the dose–response effect could not be monitored and recorded among the study participants.

However, our study also has some important strengths: (1) the analysis of the combination of CoQ10 plus NADH as a nutritional supplement in ME/CFS, with the inclusion of a substantial number of participants, (2) the improvement in the perception of cognitive fatigue and health-related quality of life achieved by the combination of CoQ10 plus NADH, (3) the homogeneous inclusion criteria through the 1994 CDC/Fukuda definition, and (4) the consideration of gender with the inclusion only of women—the recruitment of age-and sex-matched participants is essential in mitochondrial function studies, due to age-related mitochondrial function decline (such as uncoupled mitochondrial respiration, citrate synthase activity and ATP levels)—and (5) the demonstration of the safety and good tolerance of the CoQ10 plus NADH administration among participants.

Multicenter trials are now required with homogeneous patient samples after carrying out the correct clinical phenotyping (through the establishment of well-defined subgroups, based on the diagnostic criteria, neurological symptoms, comorbid health conditions, and pharmacological and non-pharmacological treatments administered), and with recommendations to eat a balanced diet. We also support long-term follow-up studies including objective physiological and biological indicators, such as peak oxygen consumption (VO_2_ as “gold standard” measure) and lactate/glucose ratio following an ergospirometry provocation test, and the assessment of information processing speed using computerized neuropsychological tests in order to verify the potential benefits of antioxidant therapy in people with ME/CFS.

## 5. Conclusions

To the best of our knowledge, this is the first study to assess the effects of oral CoQ10 plus NADH supplementation administered to a substantial number of ME/CFS patients (*n* = 207). Our findings suggest that, over a two-month period, this combination is potentially effective in reducing cognitive fatigue (also known as “brain fog”) and overall fatigue perception, thus improving HRQoL in ME/CFS. The study shows that CoQ10 and NADH can be safely co-administered to ME/CFS patients and are generally well-tolerated at the dosages indicated. A therapeutic effect was also demonstrated on sleep quality within the experimental group. Long-term RCTs in larger ME/CFS cohorts should now be performed to confirm the effectiveness of CoQ10 and NADH co-supplementation in treating the hallmark symptom of post-exertional malaise using two-day consecutive cardiopulmonary exercise testing (2-day CPET). 

## Figures and Tables

**Table 1:** Baseline demographic and clinical characteristics of study participants who completed the final assessment.

Variables	CoQ10 + NADH (*n* = 72)	Placebo (*n* = 72)	*p*-Values
Age (years)	45.38 ± 7.81	46.79 ± 6.48	0.238
BMI (kg/m^2^)	24.07 ± 4.36	24.88 ± 4.85	0.296
Systolic BP (mmHg)	124.44 ± 10.50	125.32 ± 11.85	0.639
Diastolic BP (mmHg)	75.63 ± 9.33	75.54 ± 8.21	0.954
HR (bpm)	78.96 ± 9.42	77.51 ± 10.78	0.393
Symptom onset (years)	36.94 ± 8.98	36.65 ± 7.95	0.836
Illness duration (months)	97.21 ± 69.40	106.56 ± 74.55	0.437
Concomitant drugs			
Anticonvulsants	27 (37.5)	27 (37.5)	1.000
Tricyclic antidepressants	34 (47.2)	38 (52.8)	0.505
Anxiolytics/hypnotics	14 (19.4)	18 (25.0)	0.422
NSAIDs	48 (66.7)	49 (68.1)	0.858
Opioids	15 (20.8)	24 (33.3)	0.091

Data are expressed as means ± standard deviation (SD) for continuous variables and compared by Student’s *t*-test, and as numbers of participants with percentages (%) for categorical variables and compared by Chi-squared test. Abbreviations: BMI, body mass index; BP, blood pressure; HR, heart rate; NSAIDs, non-steroidal anti-inflammatory drugs.

**Table 2:** Fatigue severity (FIS-40 questionnaire) scores of the participants completing the final assessment.

FIS-40 Domains	CoQ10 + NADH (*n* = 72)	Placebo (*n* = 72)	*p*-Values ^1^
Physical functioning			
Baseline	35.31 ± 4.61	34.82 ± 5.85	0.580
4 weeks	34.51 ± 5.39	34.14 ± 6.57	0.708
8 weeks	34.74 ± 5.21	34.42 ± 5.52	0.721
4 weeks post-treatment	35.71 ± 5.26	36.64 ± 5.43	0.554
Cognitive			
Baseline	34.26 ± 5.26	32.97 ± 7.12	0.217
4 weeks	33.14 ± 5.94 **	31.83 ± 7.07	0.232
8 weeks	33.13 ± 6.28 **	32.40 ± 7.00	0.515
4 weeks post-treatment	33.44 ± 6.90	32.97 ± 6.83	0.680
Psychosocial			
Baseline	64.78 ± 11.69	62.31 ± 13.71	0.246
4 weeks	63.44 ± 12.42	61.19 ± 14.29	0.315
8 weeks	63.94 ± 12.69	61.83 ± 14.42	0.352
4 weeks post-treatment	64.79 ± 13.53	62.97 ± 14.91	0.444
Total FIS-40 scores (0–160)			
Baseline	134.35 ± 19.80	130.10 ± 25.35	0.264
4 weeks	131.10 ± 22.15 *	127.17 ± 25.89	0.329
8 weeks	131.81 ± 22.73	128.65 ± 25.60	0.435
4 weeks post-treatment	133.40 ± 24.33	130.58 ± 25.59	0.499

Data are given as means ± SD and compared by paired Student’s *t*-test for within-group comparison analysis and by Student’s *t*-test for independent data for between-group comparison analysis. Significance level was set at * *p* < 0.05 and ** *p* < 0.01. Statistically significant differences were found in the overall FIS-40 score at 4 weeks from baseline (*p* = 0.022) in the experimental group. Abbreviations: FIS-40, 40-item fatigue impact scale. Lower FIS-40 scores indicate an improvement in fatigue perception among participants. ^1^ *p*-values for between-group comparison analysis.

**Table 3:** Sleep quality (assessed with the PSQI questionnaire) in participants completing the final assessment.

PSQI Domains	CoQ10 + NADH (*n* = 72)	Placebo (*n* = 72)	*p*-Values ^1^
Subjective sleep quality			
Baseline	1.71 ± 1.14	2.01 ± 1.08	0.101
4 weeks	1.67 ± 1.15	2.01 ± 1.08	0.064
8 weeks	1.69 ± 1.23	2.03 ± 1.13	0.091
4 weeks post-treatment	1.94 ± 1.06 *	2.10 ± 1.04	0.383
Sleep latency			
Baseline	2.29 ± 0.88	2.10 ± 1.04	0.226
4 weeks	2.25 ± 0.92	2.10 ± 1.04	0.350
8 weeks	2.40 ± 0.88	2.17 ± 1.05	0.145
4 weeks post-treatment	2.33 ± 0.87	2.07 ± 1.15	0.124
Sleep duration			
Baseline	1.31 ± 1.02	1.71 ± 0.98	0.017
4 weeks	1.28 ± 1.00	1.67 ± 0.96	0.018
8 weeks	1.51 ± 1.03 *	1.75 ± 0.98	0.160
4 weeks post-treatment	1.43 ± 0.96	1.67 ± 1.02	0.155
Habitual sleep efficiency			
Baseline	1.81 ± 1.25	2.03 ± 1.15	0.269
4 weeks	1.68 ± 1.25	1.94 ± 1.19	0.196
8 weeks	1.90 ± 1.13 *	1.97 ± 1.20	0.720
4 weeks post-treatment	1.94 ± 1.11	1.93 ± 1.23	0.943
Sleep disturbances			
Baseline	2.35 ± 0.70	2.35 ± 0.63	1.000
4 weeks	2.31 ± 0.76	2.35 ± 0.63	0.721
8 weeks	2.31 ± 0.64	2.33 ± 0.75	0.811
4 weeks post-treatment	2.36 ± 0.61	2.32 ± 0.65	0.691
Sleeping medication use			
Baseline	1.93 ± 1.23	1.75 ± 1.36	0.404
4 weeks	1.90 ± 1.25	1.75 ± 1.36	0.483
8 weeks	1.96 ± 1.22	1.88 ± 1.36	0.699
4 weeks post-treatment	1.99 ± 1.14	1.81 ± 1.34	0.385
Daytime dysfunction			
Baseline	2.15 ± 0.85	2.15 ± 0.88	1.000
4 weeks	2.13 ± 0.87	2.15 ± 0.88	0.849
8 weeks	2.18 ± 0.79	2.19 ± 0.91	0.922
4 weeks post-treatment	2.22 ± 0.74	2.22 ± 084	1.000
Global PSQI score			
Baseline	14.71 ± 3.47	15.07 ± 3.48	0.533
4 weeks	14.44 ± 3.86	15.03 ± 3.47	0.341
8 weeks	15.03 ± 3.44	15.35 ± 3.70	0.592
4 weeks post-treatment	15.26 ± 3.09	15.18 ± 3.71	0.883

Data are given as means ± SD and compared by paired Student’s *t*-test for within-group comparison analysis and by Student’s *t*-test for independent data in between-group comparison analysis. Significance level was set at * *p* < 0.05. No statistically significant differences were displayed in any PSQI items between groups. Abbreviations: PSQI, Pittsburgh sleep quality index. Lower PSQI scores indicate an improvement in the perception of sleep quality among participants. ^1^ *p*-values for between-group comparison analysis.

**Table 4:** Health-related quality of life (SF-36 questionnaire) in participants completing the final assessment.

SF-36 Domains	CoQ10 + NADH (*n* = 72)	Placebo (*n* = 72)	*p*-Values ^1^
Physical functioning			
Baseline	25.28 ± 15.72	28.47 ± 18.49	0.266
4 weeks	27.50 ± 17.78 *	30.26 ± 19.70	0.378
8 weeks	29.58 ± 18.98 **	30.35 ± 20.02	0.814
4 weeks post-treatment	28.89 ± 20.70	32.64 ± 21.18	0.284
Physical role functioning			
Baseline	3.47 ± 15.31	3.82 ± 15.51	0.892
4 weeks	4.86 ± 17.12	6.31 ± 21.28	0.654
8 weeks	5.56 ± 17.91	6.60 ± 19.68	0.740
4 weeks post-treatment	6.94 ± 22.29	2.43 ± 10.41	0.121
Bodily pain			
Baseline	15.42 ± 13.68	17.44 ± 14.04	0.381
4 weeks	18.49 ± 15.80 *	18.17 ± 15.50	0.902
8 weeks	18.24 ± 14.61	17.44 ± 17.58	0.769
4 weeks post-treatment	17.58 ± 16.07	18.60 ± 14.07	0.687
General health perception			
Baseline	24.08 ± 15.70	18.36 ± 12.55	0.017
4 weeks	21.79 ± 15.10	18.65 ± 13.53	0.191
8 weeks	21.72 ± 16.44	19.08 ± 13.39	0.292
4 weeks post-treatment	21.93 ± 17.27	17.08 ± 12.35	0.054
Vitality			
Baseline	17.64 ± 17.98	16.32 ± 16.27	0.645
4 weeks	17.08 ± 16.80	16.33 ± 15.95	0.783
8 weeks	16.39 ± 16.43	18.33 ± 19.70	0.521
4 weeks post-treatment	15.28 ± 18.04	13.82 ± 13.93 *	0.588
Social role functioning			
Baseline	30.21 ± 24.35	30.38 ± 23.44	0.965
4 weeks	28.82 ± 24.34	32.32 ± 23.95	0.386
8 weeks	28.13 ± 24.08	32.12 ± 23.72	0.317
4 weeks post-treatment	28.47 ± 23.00	29.69 ± 22.64	0.749
Emotional role functioning			
Baseline	36.11 ± 47.39	36.57 ± 43.75	0.951
4 weeks	36.57 ± 46.19	37.06 ± 43.87	0.948
8 weeks	32.87 ± 43.87	34.26 ± 42.23	0.846
4 weeks post-treatment	29.63 ± 43.89	28.24 ± 39.42	0.842
Mental health status			
Baseline	46.50 ± 21.87	45.67 ± 19.91	0.811
4 weeks	43.89 ± 21.26	47.13 ± 21.80	0.368
8 weeks	45.06 ± 22.43	47.44 ± 19.03	0.491
4 weeks post-treatment	44.72 ± 22.92	44.17 ± 21.37	0.880

Data are given as means ± SD and compared by paired Student’s *t*-test for within-group comparison analysis and by Student’s *t*-test for independent data for between-group comparison analysis. Significance level was set at * *p* < 0.05, and ** *p* < 0.01. No statistically significant differences were found in any SF-36 items in between-group comparison over time. Abbreviations: SF-36, 36-item short form health survey. Higher SF-36 scores indicate better health-related quality of life among participants. ^1^ *p*-values for between-group comparison analysis.

## Data Availability

All relevant data analyzed during the current trial are included in the article. Access to raw datasets may be provided upon reasonable request to the corresponding author.
